# Re-irradiation for recurrent glioma- the NCI experience in tumor control, OAR toxicity and proposal of a novel prognostic scoring system

**DOI:** 10.1186/s13014-017-0930-9

**Published:** 2017-11-29

**Authors:** Andra Valentina Krauze, Cord Peters, Jason Cheng, Holly Ning, Megan Mackey, Lindsay Rowe, Theresa Cooley-Zgela, Dee Dee Smart, Kevin Camphausen

**Affiliations:** Radiation Oncology Branch, Center for Cancer Research, National Cancer Institute, NIH, 9000 Rockville Pike, Building 10, CRC, Bethesda, MD 20892 USA

## Abstract

**Purpose/objectives:**

Despite mounting evidence for the use of re-irradiation (re-RT) in recurrent high grade glioma, optimal patient selection criteria for re-RT remain unknown. We present a novel scoring system based on radiobiology principles including target independent factors, the likelihood of target control, and the anticipated organ at risk (OAR) toxicity to allow for proper patient selection in the setting of recurrent glioma.

**Materials/methods:**

Thirty one patients with recurrent glioma who received re-RT (2008–2016) at NCI – NIH were included in the analysis. A novel scoring system for overall survival (OS) and progression free survival (PFS) was designed to include:1) target independent factors (age, KPS (Karnofsky Performance Status), histology, presence of symptoms), 2) target control, and 3) OAR toxicity risk. Normal tissue complication probability (NTCP) calculations were performed using the Lyman model. Kaplan-Meier analysis was performed for overall survival (OS) and progression free survival (PFS) for comparison amongst variables.

**Results:**

No patient, including those who received dose to OAR above the published tolerance dose, experienced any treatment related grade 3–5 toxicity with a median PFS and OS from re-RT of 4 months (0.5–103) and 6 months (0.7–103) respectively. Based on cumulative maximum doses the average NTCP was 25% (0–99%) for the chiasm, 21% (0–99%) for the right optic nerve, 6% (0–92%) for the left optic nerve, and 59% (0–100%) for the brainstem. The independent factor and target control scores were each statistically significant for OS and the combination of independent factors plus target control was also significant for both OS (*p* = 0.02) and PFS (*p* = 0.006). The anticipated toxicity risk score was not statistically significant.

**Conclusion:**

Our scoring system may represent a novel approach to patient selection for re-RT in recurrent high grade glioma. Further validation in larger patient cohorts including compilation of doses to tumor and OAR may help refine this further for inclusion into clinical trials and general practice.

**Electronic supplementary material:**

The online version of this article (10.1186/s13014-017-0930-9) contains supplementary material, which is available to authorized users.

## Background

Survival following concurrent radiation (RT) and temozolomide (TMZ) as per the EORTC 26981/22981-NCIC CE3 trial remains poor with median survival ranging from 39 months in RPA (Recursive Partitioning Analysis) class I patients to 5.2 months in RPA class VI patients [1]. Despite improvements in the detection of distinct molecular signatures [2], increased precision in the administration of radiation therapy and the increasing availability of targeted and non-targeted systemic agents, additional improvements are still needed.

Brain tumor recurrences can be identified by the development of new neurological symptoms, radiographic changes or both [3]. Upon recurrence, the treatment recommendations can vary widely and are partially based on the patient’s performance status, tumor location, and time interval since last treatment. Depending on these factors, options may include re-resection, chemotherapy, re-irradiation (re-RT) or enrollment on a clinical trial [4]. However, tumor re-resection is possible in less than 50% of patients [5] and the response to systemic treatment, if it occurs, is short lived with overall survival (OS) from 7.1 to 9.6 months [4]. For a significant proportion of patients with recurrent glioma in whom re-resection is not possible and for whom systemic options have been exhausted, re-RT has emerged as a possible treatment option.

In multiple retrospective trials, re-RT for brain tumors has been shown to be feasible, with a median OS benefit of 8 months, a progression free survival (PFS) of 5 months and minimal reported significant toxicities [6–15]. Acute toxicities including alopecia, headaches and nausea/vomiting, were mild and well managed with medical therapy, while late CNS toxicity was reported in most retrospective studies at a rate of less than 5%.

Existing scoring systems that may help guide patient selection for re-RT have been validated in some cohorts [10] but not in others [11]. Whilst they all include some common features rated as important by glioma re-RT experts (personal communication), they also differ with respect to inclusion of resection status, recurrent tumor size, patient symptoms and steroid use.

Toxicity attribution in patients with CNS tumors is challenging as both tumor progression and symptomatic radiation necrosis can cause significant clinical decline in the setting of short survival which limits long term evaluation. The current toxicity reporting structure is the Common Terminology Criteria for Adverse Events (CTCAEv4) [16]. For the three organs at risk (OAR) involved when contemplating CNS re-RT, the optic nerves, chiasm and brainstem, the toxicity scales remain imprecise or absent all together. In addition, most re-RT for recurrent glioma is by necessity administered in hypofractionated schedules where toxicity data and the ability to estimate normal tissue complication probabilities is limited. Since 50% of superiorly selected patients can live up to 2 years following re-RT as the systemic therapies become more effective, toxicity estimation in the setting of re-RT will become increasingly important.

In this study, we examined patient outcome using patient and recurrent tumor related factors as well as OAR dose and clinical toxicity information with NTCP calculations in patients who underwent re-RT with commonly used dose fractionation schemes. Despite the lack of a clear definition of toxicity [17–19], While NTCP calculations suggest the possibility of significant toxicity in some patients, clinical data suggests minimal toxicity following re-RT. Our novel scoring system based on the ability to control recurrent tumor, risk of toxicity as well a more traditional scoring parameters (age, histology, symptomatic, disease free interval) with re-RT, may represent a step forward in the selection of patients for re-RT.

## Methods and materials

### Patients

Thirty one patients with recurrent glioma or gliosarcoma underwent re-RT, at the National Cancer Institute between 2008 and 2016. Chart review and treatment planning information including dose distributions and treatment volumes were collected from both the original and subsequent treatment plans whenever possible. CTCAE v4.0 was employed to define toxicity. The patients were originally treated for glioma or gliosarcoma using IMRT or 3D conformal technique. Dosimetric analysis for NTCP was performed if the original treatment plan could be obtained and if they received their treatment at a fraction size ≤300 cGy [20]. This was possible in 25 patients.

### NTCP model and calculation

Maximum and equivalent uniform dose (EUD) for optic chiasm, optic nerves and brain stem were extracted from ECLIPSE™ or the patient’s original treatment plan documents. NTCP calculations were performed using tolerance dose values for the uniform irradiation of critical structures based on literature data and fit by Burman [21] to the NTCP model proposed by Lyman [22]. Original and retreat gross tumor volume (GTV) and planning target volume (PTV) volumes were recorded when available. The original GTV volume was obtained from the original treatment plan and in all cases was based on the T1 gadolinium enhancing component as contoured by the treating radiation oncologist. The original PTV represents the volume treated to the highest dose and is based on the original GTV. For the NTCP calculation involving the maximum dose, the maximum dose to the OAR in the first and re-treatment were obtained, converted to account for dose per fraction, and subsequently added. For the NTCP calculation involving EUD, the mean dose to the organ between the first and re-treatment were added, after adequate contouring of the OAR in both the first and re-treatment plans.

### Scoring system

A scoring system was designed to capture the parameters that underlie the administration of re-RT (Table 1). A set of imaging independent factors (target independent score) was made up by: patient age, KPS (Karnofsky Performance Status), histology and the presence of the symptoms. The ability to control the target was based on tumor size (GTV in cm^3^), tumor recurrence location with respect to original location and the presence of diffuse disease on MRI brain. The anticipated toxicity risk with re-RT administration was evaluated as a function of the location of OAR with respect to previously treated area, the dose contribution to each OAR and disease free interval from the original RT. A score of 1–3 per variable was assigned in each category for a cumulative score ranging from 10 (worst prognosis) to 30 (best prognosis).

**Table 1:** Krauze Scoring System

Prognostics Factor	Subgroups	Value for prognostic score
Independent Factors		
Patient Age	>60 years	1
	50–60 years	2
	<50 years	3
KPS	<50%	1
	50–70%	2
	>70%	3
Histology	WHO Grade IV	1
	WHO Grade III	2
	WHO Grade II	3
Presence of symptoms	Documented neurological symptoms related to recurrence requiring steroid management	1
	Documented neurologicalsymptoms relatedto recurrence or impending neuro symptoms	2
	No neurological symptoms related to recurrence	3
Target Control		
Tumor size (GTV)	>500 cm^3^ or diffuse disease/ gliomatosis	1
	20–500 cm^3^	2
	<20 cm^3^	3
Tumor recurrencelocation withrespect to originaltreatment field (60Gy isodose line)	<1 cm away or completelywithin the original treatment field	1
	1–3 cm away	2
	>3 cm away	3
Diffuse disease present	Multiple T1 gadolinium- enhancing lesions	1
	T2 FLAIR diffuse involvement	2
	None (localized recurrence only)	3
Anticipated Toxicity Risk		
OAR location with respect to recurrence area	>1 cm away from or in the recurrence area	1
	1–3 cm away from recurrence area	2
	>3 cm away from the recurrence area	3
OAR dose contribution from original treatment^a^	<90% dose allowed as per Quantec dose constraints	1
	Within +/− 10% of dose allowed as per Quantec constraints	2
	Exceeds >10% over the Quantec constraints	3
Disease free interval from initial treatment with radiation	<1 year	1
	1–3 years	2
	>3 years	3

^a^OAR QUANTEC dose constraints: Chiasm: 55Gy, Optic Nerves: 55Gy, Brain Stem: 54 Gy. *WHO* World Health Organisation

## Results

### Patient characteristics

Thirty one patients were included in the analysis. The median age was 47y/o (18-73y), 58% were male, and 81% had a pre re-RT KPS ≥70 (Table 2). 58% of patients were initially diagnosed with a GBM with the majority located in the fronto-parietal lobes. The median disease free interval was 33.7 months (11.4–174). The location of the recurrent tumor was within the previous field in all but 6 patients. During re-RT 13 patients received concurrent systemic therapy in the form of TMZ [4], bevacizumab [5], or both [1].

**Table 2:** Patient, Tumor and Treatment Characteristics

Parameter	*N* = 31
Age (yrs), median (range)	47 (18–73)
Sex	
Male	18
Female	13
KPS at time of reRT	
≤ 50	1
50–70	5
> 70	25
Original histology	
WHO grade IV	18
WHO grade III	5
WHO grade II	7
Gliosarcoma	1
Original tumor location	
Frontal	13
Parietal	5
Temporal	3
Occipitoparietal	1
Temporoparietal	3
Frontotemporal	2
Brainstem/Cerebellum	3
Other	1
Recurrence location wrt to the 80% isodose line	
Within	25
Outside	6
Other hemisphere	1
Brainstem	2
4th ventricle/post fossa	2
Adjacent Lobe	1
DFS median (range)(months)	33.7 (11.4–174)
Tissue diagnosis prior to re-RT	22
Concurrent agent administration	13
Temozolomide alone	4
Bevacizumab alone	5
Temozolomide and Bevacizumab	1
Other	3
Management upon progression after re-RT	
Bevacizumab	8
BSC	20
Other (BCNU, study)	3

*DFS* Disease Free Survival, *BSC* Best Supportive Care, *WHO* World Health Organisation

### Treatment and volume characteristics

Twenty-five patients for whom the complete original treatment plan was available were included in the dosimetric analysis (Table 3). The original treatment dose was 60Gy (median), with one patient (brainstem tumor) treated to 55.8Gy with 66% of patients treated with IMRT. The median original GTV was 28cm^3^ (5.8-72 cm^3^), and the original PTV was 307cm^3^ (107-601 cm^3^) (*N* = 18). Re-RT dose was 30Gy (15-54Gy) with a median retreat GTV of 29cm^3^ (0.3-313 cm^3^) and PTV of 124cm^3^ (0.5–511 cm^3^) with 77% treated using IMRT. The cumulative BED between the two radiation treatments had a median of 96Gy (72–112Gy). Patients treated with hypofractionated regimens (dose/fraction >300 cGy), were not included in the NTCP analysis due to concerns over the use of the linear quadratic equation as means of dose conversion and the differences in the radiobiological effects at a higher dose per fraction [20]. They represent a patient with cerebellar sarcoma treated upfront with 35Gy in 5 fractions using Cyberknife technique, and 3 additional patients re-irradiated using SRS [1] or SRT [2] technique.

**Table 3:** Radiation Treatment Characteristics

Parameter	N = 31
Dose of original RT (Gy)	
Median	60
Range	18–60
Original RT technique	
3D conformal	10
IMRT	20
Unknown (Plan not available)	1
Original GTV volume (cm^3^)	
Median	27.9
Range	5.8–72
Original PTV volume (cm^3^)	
Mean	307
Range	107–601
Interval between original RT and Re-RT (yrs)	
Median	4.28
Range	0.95–14.5
Re-RT dose (Gy)	
Median	30
Range	15–54
Re-RT dose per fraction (Gy)	
Median	2.5
Range	1.8–15
Re-RT technique	
3D conformal	5
IMRT	23
3D conformal with IMRT boost	2
SRS	1
Retreat GTV volume (cm^3^)	
Median	29
Range	0.3–313
Retreat PTV volume (cm^3^)	
Median	124
Range	0.5–511
Cumulative BED (α/β = 2), (Gy)	*N* = 25
Median	96
Range	72–112

### NTCP calculation

**Fig. 1:**
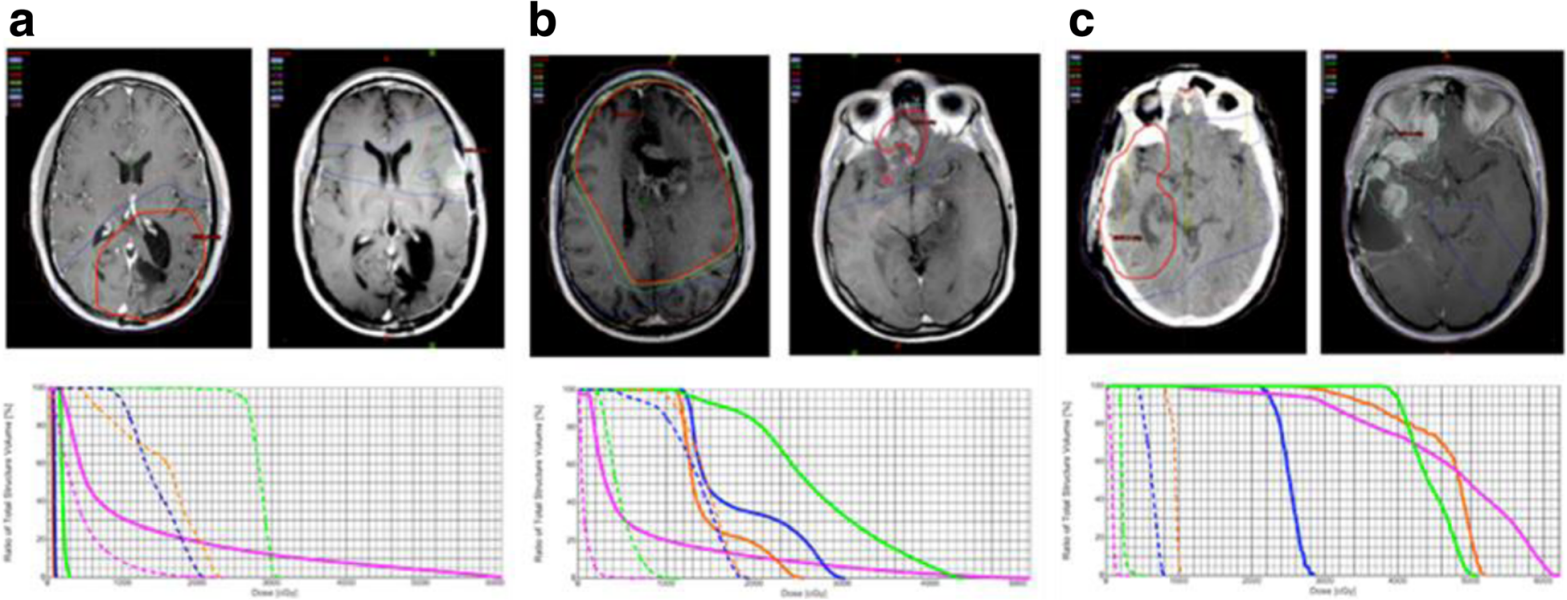
Initial treatment and reRT dose distribution with corresponding dose volume histogram (DVH) for the first (solid lines) and second (dashed lines) radiation plans. Possible scenarios are displayed: (**a**) dose to OAR lower than accepted dose constraints, (**b**) OAR barely meeting dose constraints, and (**c**) OAR above accepted constraints. Green = optic chiasm, Pink = brain stem, Blue = left optic nerve, Orange = right optic nerve

Performing NTCP calculations was possible for 25 of the re-RT patients. During the process of obtaining the volume and dose values from original and subsequent treatment plans, we noted that a significant variation can occur in how the OAR are contoured between different providers. Using CNS contouring atlases [23, 24] (IMAIOS SAS Anatomy, IMAIOS SAS, www.imaios.com, www.qarc.org/cog/ACNS0331Atlas.pdf) we re-contoured OAR structures to enable a more adequate calculation of mean dose to the organ (Fig. 1). In addition, when adding plans using the “plan sum” function in Eclipse™ inconsistent values often resulted due to changes in volume contouring, location of OAR maximal point dose and treatment technique. In order to avoid these inconsistent results we collected the max and mean values from the respective plans, converted them to account for BED, and then manually added the values (Fig. 1 and Additional file 1: Table S1A and B) as previously described in dose tolerance literature [25, 26].

### Toxicity and NTCP

Based on the cumulative maximum doses received from the first and the second courses of radiation, the average NTCP was 25% (0–99%) for the chiasm, 21% (0–99%) for the right optic nerve, 6% (0–92%) for the left optic nerve, and 59% (0–100%) for the brainstem (Additional file 2: Figure S1A and Additional file 1: Table S1A). Based on the mean (EUD) doses the average NTCP was 11% (0–91%) for the chiasm, 5% (0–50%) for the right optic nerve, 0% (0–4%) for the left optic nerve, 5% (0–92%) for the brainstem (Additional file 2: Figure S1B and Additional file 1: Table S1B). As per chart documentation and ongoing follow-up, no patient including those who received dose to OAR above the published tolerance dose, experienced any treatment related grade 3–5 toxicity. At the time of re-RT initiation, 39% of the patients were on steroids for symptom management, and an additional patient was started on steroids during re-RT. Pretreatment KPS was maintained for 45% of the patients at 1 month. The decline in KPS correlated with documentation of tumor progression outside the re-RT field in all patients. The two patients who had the highest NTCP to chiasm, optic nerves and brainstem survived 8 and 15 months from retreatment, respectively. Aside from fatigue and alopecia, no significant side effects that could be attributed to re-RT were observed.

### Scoring system

**Fig. 2:**
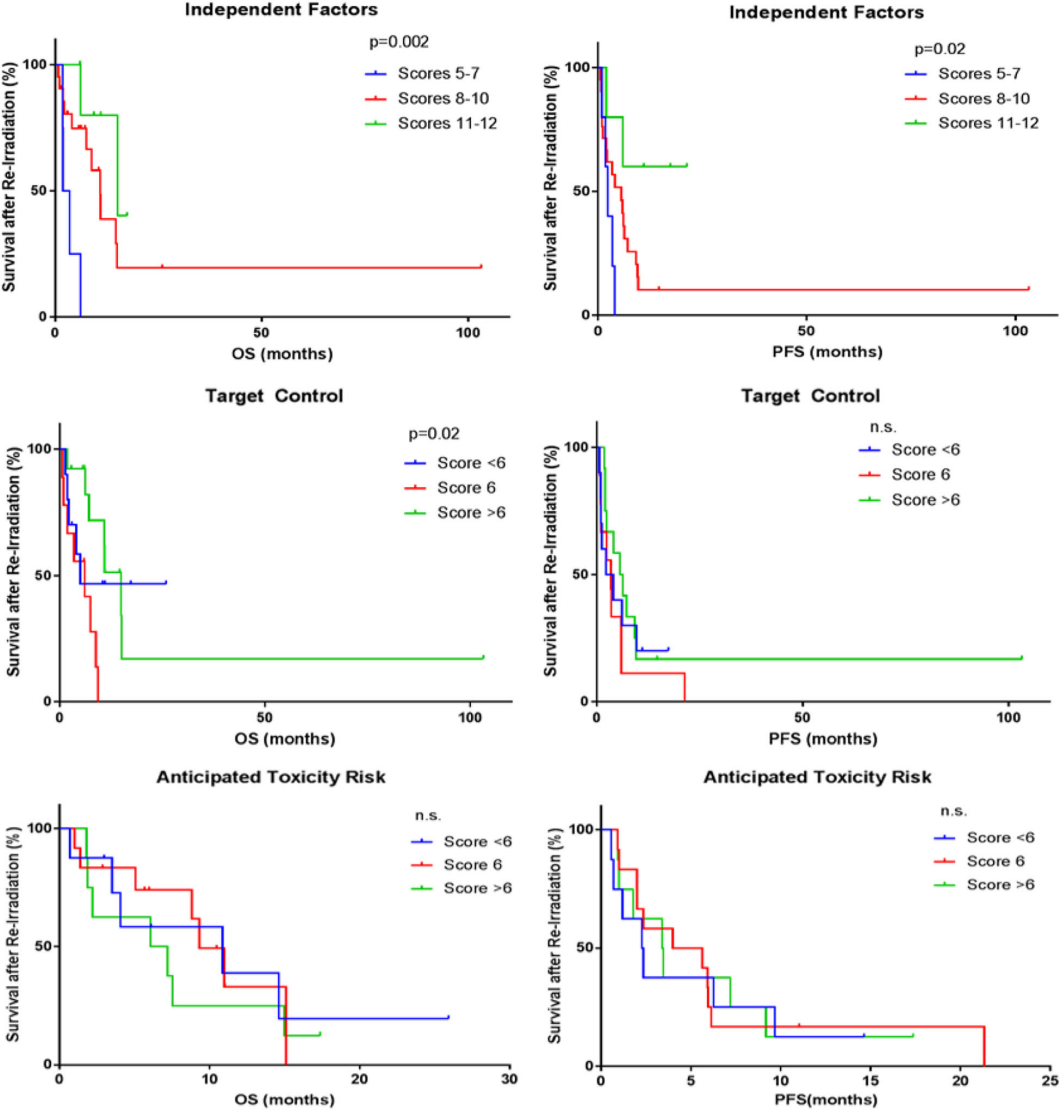
Kaplan-Meier analysis pf progression-free survival (PFS) and overall survival (OS) in re-RT patients by independent factors, anticipated target control and anticipated toxicity risk control

**Fig. 3:**
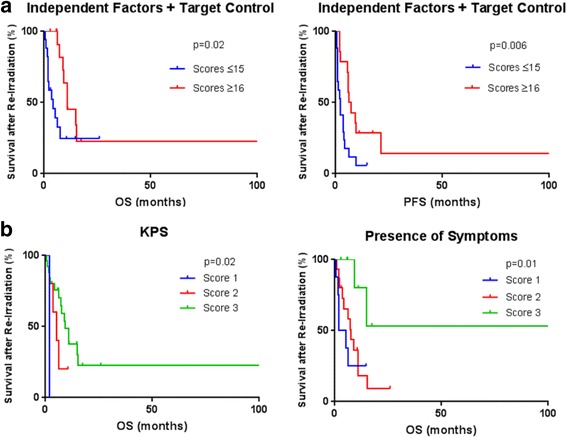
**a**. Kaplan-Meier analysis of overall survival (OS) and progression-free survival (PFS) and in re-RT patients by independent factors + target control. **b**. Kaplan-Meier analysis of overall survival (OS) by presence of symptoms and Karnofsky Performance Status (KPS)

Using the new scoring system (Table 1), we analyzed the impact of target control and the anticipated toxicity risk on OS and PFS for all re-RT patients using Kaplan-Meier analysis (Fig. 2). The independent factor subgroup was statistically significant for both OS and PFS but the target control subgroup was only significant for OS and not PFS. The anticipated toxicity risk subgroup was not statistically significant for either OS or PFS. Furthermore, the combination of the independent factor subgroup and target control subgroup was statistically significant for both OS and PFS (Fig. 3). Moreover, the presence of symptoms prior to re-RT and KPS were statistically significant for OS. Thus, using clinical parameters (age, KPS, histology and symptoms) in combination with tumor radiobiological parameters (volume, total dose, unifocal) a modified scoring system has been derived that may stratify patients better than the currently available scoring systems.

## Discussion

With the use of advanced therapy techniques and careful treatment planning, for the most part, physicians are able to minimize dose to previously radiated OAR allowing safe re-RT of brain tumor recurrences with an improvement in OS and PFS (6–15). According to the nomogram developed by Combs et al., 50% of patients with a score of 2 (i.e. GBM patients <50 year old) survive at least another 12 months from the time of re-RT [1]. Thus, a more thorough understanding of the potential toxicities that may occur after tumor re-RT are needed. Although acute toxicities in the form of alopecia, headaches and nausea/vomiting have been reported, they are self-limited and controlled with medical management. Likewise, late CNS toxicity remains underreported but in most retrospective studies is less than 5%. In our cohort of 31 patients we had no grade 3, 4 or 5 acute or late toxicities with a median PFS of 4 months (range 0.5–103 months) and OS of 6 months (range 0.7–103 months), a range in keeping with both existing validated prognostic scores [1, 11] and retrospective data.

When working on a re-RT plan for a CNS patient, the physician can use two normal tissue dose reports Emami, 1991 [27] and Quantec 2010 [17–19] to help them decide the safety of the proposed treatment as well as the side effect profile to report to the prospective patient. Additionally, mathematical models using the Lyman–Kutcher–Burman NTCP [21] based on dose have been implemented in an attempt to calculate NTCP [28]. However, several problems arise with toxicity attribution. These include the lack of reliable toxicity estimation, the relatively short PFS and OS times, the overlapping symptoms of re-RT toxicity and tumor recurrence and the concurrent or adjuvant administration of systemic agents that can alter radioresponse and toxicity measurements. Our analysis suggests that the Emami and Quantec papers and the NCTP calculations appear to overestimate the rates of clinical toxicity.

To our knowledge ours is the only paper examining NTCP and cumulative dose in the setting of re-RT for gliomas. Calculation of the cumulative dose to OAR by creating a “plan sum” for the two treatment plans was found to often result in erroneous cumulative dose to OAR hence manual summation of dose delivered was employed to calculate NTCP in this study. Nonetheless, the calculated NTCP using maximum doses to OAR between the two different treatment plans was as high as 100% in the case of all four major OAR. An expected difference in NTCP was observed when mean dose (EUD) [29] versus max dose were employed for its calculation. Considering the lack of toxicity observed, we propose that NTCP calculations based on EUD may represent a more accurate estimation of risk. Mean dose may be both more accurate and more realistic [30, 31] due to its decreased dependency on biologic parameters, including alpha/beta ratio. This raises the question as to whether the OAR in question behaves as serial, parallel or serial-parallel in terms of toxicity and this is yet unclear [32, 33].

Additionally, neither dose per fraction nor time between the two radiation treatments, or the use of concurrent agents all of which may play a significant role in the development of treatment related toxicity, are currently accounted for when calculating NTCP. The impact of concurrent chemotherapy is unclear in the re-RT setting and may further defined by results of ongoing trialssuch as RTOG1205) which explores concurrent bevacizumab, NCT02709226 (Krauze et al.) allowing both concurrent temozolomide and bevacizumab and upcoming trials that explore concurrent temozolomide. Furthermore, most patients who receive re-RT tend to be younger with superior performance status and patient age has been shown to have some correlation with the development of toxicity or lack thereof [17]. This is an important factor when considering the lack of toxicity noted in this study. Limitations to our data include long recruiting times for the patient cohort and inability to carry out NTCP calculations in all patients Long recruiting times for the patient cohort add to the heterogeneity of the data, they are however unavoidable as patients who are referred for re-RT are often referred by virtue of the fact that they have a long disease free interval and a change in the treatment technique is therefore also more likely as technology evolves. However, our data does not suggest that technique played a role. We did note that patients who received 3D conformal RT the first time were more likely to receive IMRT on re-RT in order to decrease dose to OAR. NCTP calculations were only possible for 25 of 31 patients due to the following limitations: 1) inability to obtain the first radiation treatment plan often due to a longer time period since previous treatment (>5 years) or loss of the previous planning data and 2) use of a non conventional fractionation scheme, ie a hypofractionated scheme wherein usage of the linear quadratic equation for BED calculation is not generally accepted [20]. This does limit the results of the study in that it reduced the overall data available, however overall the results obtained do reflect a more homogenous set of dose fractionation schemes, which are employed in ongoing and upcoming prospective trials. It is likely that the inclusion of patients who were treated with hypofractionated schemes would potentially alter the conclusions since a higher level of late toxicity may be postulated. Of note, previous treatment plans are often labor intensive to difficult to obtain and integrate with the re-irradiation plan and thus re-irradiation may be practiced in the community without evaluation of the previous plan raising the issue of cumulative dose to OAR and hence the lack of retrospective data to produce superior models for NTCP in the re-RT setting.

The identification of true toxicity and its relationship to dose, will require 1) greater reporting of dose to OAR after re-RT and 2) robust testing for potential OAR toxicity, including visual field testing, audiology, neurocognitive function and quality of life assessment. Baseline visual field testing although inconsistently obtained is more commonly carried out and thus may more readily provide information on OAR toxicity in the short term. However, visual evoked potentials may represent a more accurate modality of assessing toxicity to the visual apparatus and bears consideration for inclusion in prospective clinical trials [32]. The lack of such data in the literature delegates estimation of risk to retrospective studies and models that are inadequate thus, making already challenging patient discussions and decision making, even more so.

When applying either the Carson 2007 [34] or the Combs 2013 [1] scoring system to our data, we did not achieve a statistically significant separation of the curves (Additional file 3: Figure S2) as has been noted in other published dataset comparisons [9], this however may reasonably be related to the small sample size in our study as compared to that of other authors Unlike the Carson 2007 scoring system, the Combs 2013 scoring system does not include KPS or steroid use but does include time since previous RT in addition to age and histology. Our scoring system considers the likelihood of controlling the recurrent tumor (target control) based on tumor size, tumor location and presence of diffuse disease parameters derived from our own practice and review of the practice and that of other radiation prominent oncologists (personal communication). In our analysis, the tumor control subgroup was highly significant for OS. In addition, we have shown that while KPS matters in terms of OS with re-RT, it is the patient’s symptomatic or asymptomatic status, which may be a more important surrogate of both PFS and OS. This indicates that parameters, currently not included in existing scoring systems may represent reasonable additions to a universally validated scoring system. The use of steroids is a challenging parameter for OS or PFS or for that matter as a study endpoint, as a significant proportion of patients is on steroids prior to re-RT, some for symptoms, others prophylactically. We did not find steroid use to be a useful parameter in our study. Steroid use may be helpful although the heterogeneity of its use in terms of doses, dose increases and the inconsistent capture of steroid usage details may make its consistent comparison and inclusion in scoring systems challenging.

This scoring system was developed out of the need to find a better way to select optimal patients for re-RT who stand to gain palliative benefit and for whom the risk of re-RT is considered acceptable. Further validation in larger cohorts will be required to validate and refine target based scoring systems such as this one.

## Conclusion

No treatment related complications were observed in re-RT patients included in our study. Existing NTCP calculations based on maximal point dose and mean dose (EUD) to OAR may overestimate NTCP in the re-RT of high grade glioma. Ongoing heterogeneity in the approach to patient selection for re-RT underscores the need for a universally validated tumor and patient characteristics based scoring system.

## Additional files

Additional file 1: Table S1. *A. maximum* Dose and NTCP to OAR based on Maximum Dose values. B. Mean Dose (EUD) and NTCP to OAR based on Mean Dose values. (DOCX 200 kb)

Additional file 2: Figure S1. Normal Tissue complication probability (NTCP) (%) vs. *A. maximum* dose administered to the organ. B. Mean Dose administered to the organ. TheTD65/5 (Maximum Tolerated Dose 50% rate at 5 years at a dose of 65 Gy) curve based on Emami et al. was used to model NTCP using our retrospective data. (DOCX 58 kb)

Additional file 3: Figure S2. Comparison with existing scoring systems A. Carson 2007. B. Combs 2013. (DOCX 28 kb)

## Abbreviations

BED: Biologically Equivalent Dose
CNS: Central Nervous System
CTCAE: Common Terminology Criteria for Adverse Events
EORTC: European Organisation for Research and Treatment of Cancer
EUD: Equivalent Uniform Dose
GBM: Glioblastoma
GTV: Gross Tumour Volume
IMRT: Intensity Modulated Radiation Therapy
KPS: Karnofsky Performance Status
MRI: Magnetic Resonance Imaging
NCI: National Cancer Institute
NTCP: Normal Tissue Complication Probability
OAR: Organs at Risk in the Field
OS: Overall Survival
PFS: Progression Free Survival
PTV: Planning Treatment Volume
Re-RT: Re-irradiation
RPA: Recursive Partitioning Analysis
RT: Radiation Therapy
SRS: Stereotactic Radiosurgery
SRT: Stereotactic Radiotherapy
TMZ: Temozolomide
