# Supplementary material for: Re-irradiation for recurrent glioma- the NCI experience in tumor control, OAR toxicity and proposal of a novel prognostic scoring system
Source: Radiat Oncol. 2017 Nov 29;12:191. doi: 10.1186/s13014-017-0930-9 (PMC5707810; doi:10.1186/s13014-017-0930-9)
Supplement: Supplementary file 1 — A. maximum Dose and NTCP to OAR based on Maximum Dose values. B. Mean Dose (EUD) and NTCP to OAR based on Mean Dose values. (DOCX 200 kb) [file 13014_2017_930_MOESM1_ESM.docx]

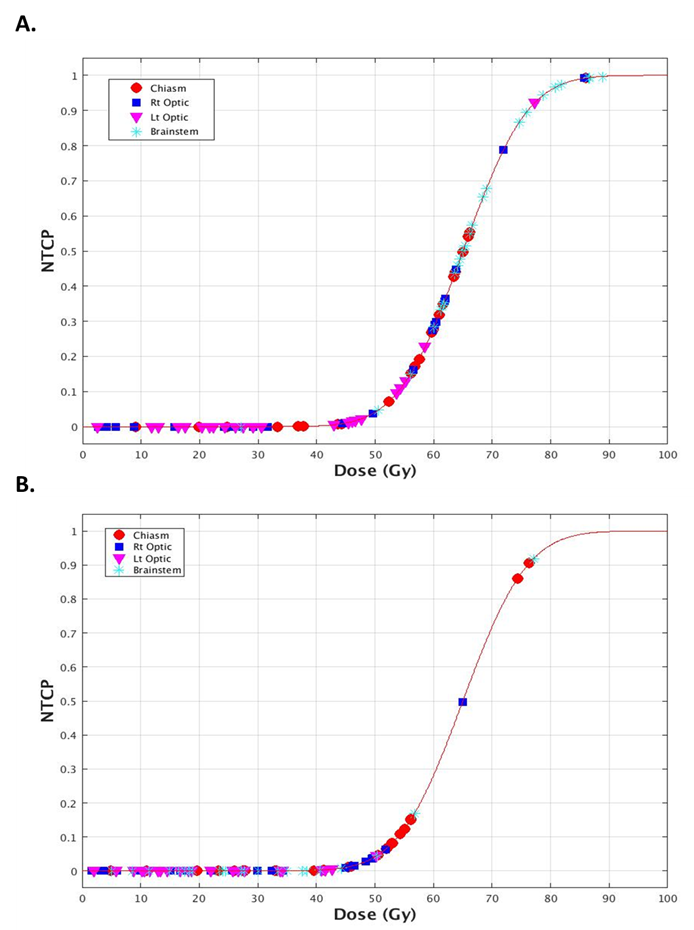


**Supplemental Figure 1.** Normal Tissue complication probability (NTCP) (%) vs. **A.** Maximum dose administered to the organ. **B.** Mean Dose administered to the organ. TheTD65/5 (Maximum Tolerated Dose 50% rate at 5 years at a dose of 65 Gy) curve based on Emami et al. was used to model NTCP using our retrospective data.
