# Supplementary figures and images for: Re-irradiation for recurrent glioma- the NCI experience in tumor control, OAR toxicity and proposal of a novel prognostic scoring system
Source: Radiat Oncol. 2017 Nov 29;12:191. doi: 10.1186/s13014-017-0930-9 (PMC5707810; doi:10.1186/s13014-017-0930-9)

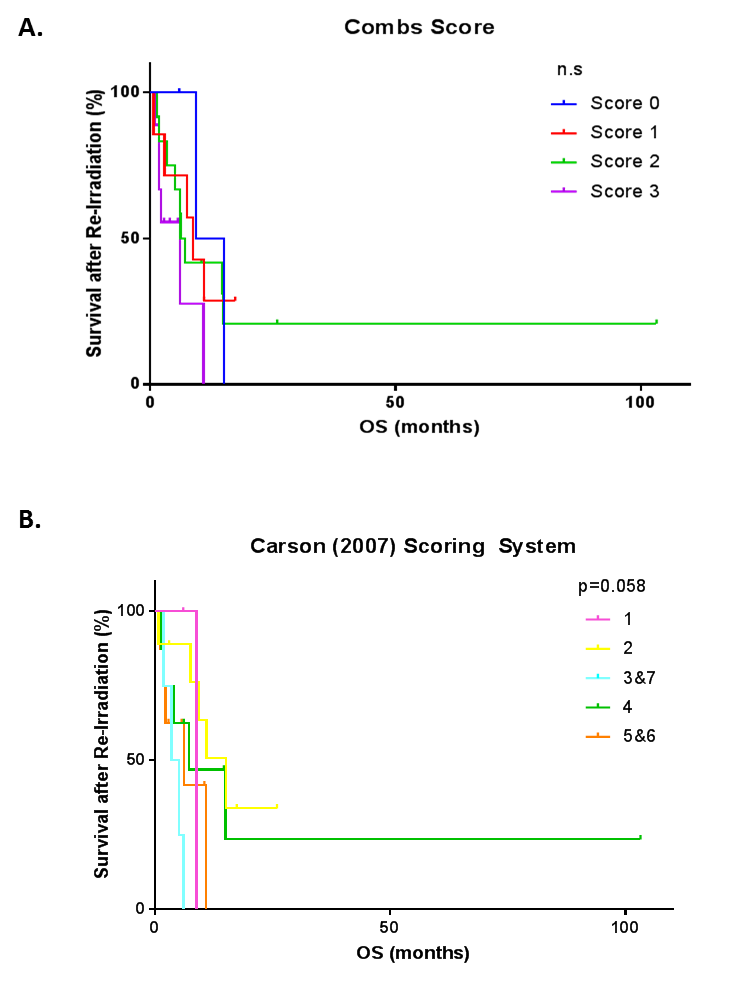


**Supplemental Figure 2**. Comparison with existing scoring systems **A.** Combs 2013. **B.** Carson 2007.

Supplement: Supplementary file 2 — Normal Tissue complication probability (NTCP) (%) vs. A. maximum dose administered to the organ. B. Mean Dose administered to the organ. TheTD65/5 (Maximum Tolerated Dose 50% rate at 5 years at a dose of 65 Gy) curve based on Emami et al. was used to model NTCP using our retrospective data. (DOCX 58 kb) [file 13014_2017_930_MOESM2_ESM.docx]
