# Supplementary material for: Re-irradiation for recurrent glioma- the NCI experience in tumor control, OAR toxicity and proposal of a novel prognostic scoring system
Source: Radiat Oncol. 2017 Nov 29;12:191. doi: 10.1186/s13014-017-0930-9 (PMC5707810; doi:10.1186/s13014-017-0930-9)
Supplement: Supplementary file 3 — Comparison with existing scoring systems A. Carson 2007. B. Combs 2013. (DOCX 28 kb) [file 13014_2017_930_MOESM3_ESM.docx]

**Supplemental Table 1. A.** Maximum Dose and Normal Tissue Complication Probability (NTCP) to organs at risk (OAR) based on Maximum Dose values.

|  | Chiasm | | Right optic nerve | | Left optic nerve | | Brainstem | |
| --- | --- | --- | --- | --- | --- | --- | --- | --- |
|  | **Dose (Gy)** | **NTCP (%)** | **Dose (Gy)** | **NTCP (%)** | **Dose (Gy)** | **NTCP (%)** | **Dose**  **(Gy)** | **NTCP (%)** |
| 1 | 36.8 | 0.1 | 26.4 | 0.0 | 24.3 | 0.0 | 86.6 | 99.4 |
| 2 | 57.5 | 19.3 | 49.6 | 3.8 | 53.7 | 9.5 | 61.7 | 35.3 |
| 3 | 9.0 | 0.0 | 2.6 | 0.0 | 2.6 | 0.0 | 27.1 | 0.0 |
| 4 | 56.8 | 17.1 | 44.4 | 0.9 | 21.7 | 0.0 | 62.6 | 33.2 |
| 5 | 60.9 | 31.8 | 15.7 | 0.0 | 29.1 | 0.0 | 81.8 | 97.4 |
| 6 | 43.6 | 0.7 | 4.1 | 0.0 | 12.9 | 0.0 | 61.6 | 34.6 |
| 7 | 44.3 | 0.8 | 26.0 | 0.0 | 30.6 | 0.0 | 88.8 | 99.7 |
| 8 | 65.9 | 54.2 | 63.8 | 44.6 | 26.2 | 0.0 | 69.0 | 67.9 |
| 9 | 37.7 | 0.1 | 24.2 | 0.0 | 20.4 | 0.0 | 86.7 | 99.4 |
| 10 | 24.7 | 0.0 | 15.7 | 0.0 | 17.5 | 0.0 | 60.1 | 28.3 |
| 11 | 59.7 | 26.9 | 62.0 | 36.4 | 42.9 | 0.5 | 78.7 | 94.4 |
| 12 | 66.1 | 55.3 | 71.9 | 78.7 | 46.1 | 1.4 | 74.6 | 86.7 |
| 13 | 63.4 | 42.6 | 60.2 | 29.0 | 45.4 | 1.2 | 64.1 | 45.8 |
| 14 | 59.7 | 26.9 | 56.5 | 16.2 | 55.2 | 12.9 | 64.5 | 47.6 |
| 15 | 63.7 | 44.2 | 5.6 | 0.0 | 16.3 | 0.0 | 63.6 | 43.6 |
| 16 | 56.1 | 15.3 | 24.6 | 0.0 | 27.4 | 0.0 | 66.1 | 55.2 |
| 17 | 59.8 | 27.6 | 62.0 | 36.5 | 42.9 | 0.5 | 75.8 | 89.5 |
| 18 | 66.1 | 55.3 | 71.9 | 78.7 | 46.1 | 1.4 | 74.6 | 86.7 |
| 19 | 64.9 | 49.7 | 61.8 | 35.7 | 46.6 | 1.7 | 65.3 | 51.5 |
| 20 | 52.3 | 7.1 | 29.1 | 0.0 | 54.4 | 10.9 | 66.6 | 57.3 |
| 21 | 85.9 | 99.2 | 85.7 | 99.2 | 77.3 | 92.2 | 50.5 | 4.7 |
| 22 | 59.7 | 26.9 | 60.4 | 29.7 | 58.5 | 22.7 | 80.8 | 96.6 |
| 23 | 19.9 | 0.0 | 8.7 | 0.0 | 11.8 | 0.0 | 56.1 | 15.1 |
| 24 | 33.4 | 0.0 | 31.6 | 0.0 | 22.4 | 0.0 | 65.0 | 50.2 |
| 25 | 61.6 | 34.9 | 59.8 | 27.5 | 47.6 | 2.2 | 68.4 | 65.3 |
| Average | **52.4** | **25.4** | **41.0** | **20.7** | **35.2** | **6.3** | **68.0** | **59.4** |
| Range | **9.0-85.9** | **0-99.2** | **2.6-85.7** | **0-99.2** | **2.6-77.3** | **0-92.2** | **27.1-88.8** | **0-99.7** |

**Supplemental Table 1.** **B.** Mean Dose (EUD) and Normal Tissue Complication Probability (NTCP) to organs at risk (OAR) based on Mean Dose values.

|  | Chiasm | | Right optic nerve | | Left optic nerve | | Brainstem | |
| --- | --- | --- | --- | --- | --- | --- | --- | --- |
|  | **Dose (Gy)** | **NTCP (%)** | **Dose (Gy)** | **NTCP (%)** | **Dose (Gy)** | **NTCP (%)** | **Dose (Gy)** | **NTCP (%)** |
| 1 | 32.9 | 0.0 | 17.4 | 0.0 | 16.8 | 0.0 | 16.5 | 0.0 |
| 2 | 33.2 | 0.0 | 32.4 | 0.0 | 34.2 | 0.0 | 8.5 | 0.0 |
| 3 | 4.9 | 0.0 | 1.7 | 0.0 | 2.0 | 0.0 | 5.0 | 0.0 |
| 4 | 52.1 | 6.7 | 34.4 | 0.0 | 8.8 | 0.0 | 37.6 | 0.1 |
| 5 | 46.0 | 1.4 | 9.5 | 0.0 | 13.0 | 0.0 | 77.1 | 91.9 |
| 6 | 23.3 | 0.0 | 3.2 | 0.0 | 11.6 | 0.0 | 15.0 | 0.0 |
| 7 | 27.8 | 0.0 | 15.1 | 0.0 | 18.2 | 0.0 | 30.0 | 0.0 |
| 8 | 50.5 | 4.7 | 51.8 | 6.4 | 18.7 | 0.0 | 27.2 | 0.0 |
| 9 | 27.4 | 0.0 | 17.1 | 0.0 | 13.4 | 0.0 | 33.4 | 0.0 |
| 10 | 19.6 | 0.0 | 10.3 | 0.0 | 10.7 | 0.0 | 23.4 | 0.0 |
| 11 | 52.9 | 8.1 | 29.9 | 0.0 | 22.0 | 0.0 | 40.6 | 0.2 |
| 12 | 56.1 | 15.2 | 64.9 | 49.7 | 40.9 | 0.3 | 56.7 | 16.9 |
| 13 | 51.9 | 6.5 | 44.9 | 1.0 | 26.7 | 0.0 | 24.1 | 0.0 |
| 14 | 54.3 | 10.9 | 49.6 | 3.8 | 42.7 | 0.5 | 44.3 | 0.8 |
| 15 | 39.6 | 0.2 | 3.7 | 0.0 | 10.2 | 0.0 | 17.6 | 0.0 |
| 16 | 41.3 | 0.3 | 16.7 | 0.0 | 13.4 | 0.0 | 27.7 | 0.0 |
| 17 | 52.9 | 8.1 | 29.9 | 0.0 | 22.0 | 0.0 | 40.6 | 0.2 |
| 18 | 56.1 | 15.1 | 64.9 | 49.7 | 40.9 | 0.3 | 56.7 | 16.9 |
| 19 | 53.0 | 8.3 | 46.2 | 1.5 | 27.7 | 0.0 | 24.5 | 0.0 |
| 20 | 76.3 | 90.5 | 25.6 | 0.0 | 25.7 | 0.0 | 38.4 | 0.1 |
| 21 | 74.4 | 86.1 | 49.5 | 3.7 | 50.2 | 4.3 | 18.4 | 0.0 |
| 22 | 45.4 | 1.2 | 48.4 | 2.8 | 41.5 | 0.3 | 50.4 | 4.6 |
| 23 | 11.0 | 0.0 | 5.9 | 0.0 | 5.8 | 0.0 | 11.4 | 0.0 |
| 24 | 26.0 | 0.0 | 22.0 | 0.0 | 14.6 | 0.0 | 18.8 | 0.0 |
| 25 | 55.0 | 12.4 | 46.5 | 1.6 | 33.8 | 0.0 | 34.9 | 0.0 |
| Average | **42.6** | **11.0** | **29.7** | **4.8** | **22.6** | **0.2** | **31.2** | **5.3** |
| Range | **4.9-76.3** | **0-90.5** | **1.7-64.9** | **0-49.7** | **2-50.2** | **0-4.3** | **8.5-77.1** | **0-91.9** |
